# Sensor and Sensor Fusion Technology in Autonomous Vehicles: A Review

**DOI:** 10.3390/s21062140

**Published:** 2021-03-18

**Authors:** De Jong Yeong, Gustavo Velasco-Hernandez, John Barry, Joseph Walsh

**Affiliations:** 1 IMaR Research Centre, Munster Technological University, V92 CX88 Tralee, Ireland; gustavo.velascohernandez@staff.ittralee.ie (G.V.-H.); john.barry@staff.ittralee.ie (J.B.); joseph.walsh@staff.ittralee.ie (J.W.); 2 School of Science Technology, Engineering and Mathematics, Munster Technological University, V92 CX88 Tralee, Ireland; 3 Lero—Science Foundation Ireland Research Centre for Software, V92 NYD3 Limerick, Ireland

**Keywords:** autonomous vehicles, self-driving cars, perception, camera, lidar, radar, sensor fusion, calibration, obstacle detection

## Abstract

With the significant advancement of sensor and communication technology and the reliable application of obstacle detection techniques and algorithms, automated driving is becoming a pivotal technology that can revolutionize the future of transportation and mobility. Sensors are fundamental to the perception of vehicle surroundings in an automated driving system, and the use and performance of multiple integrated sensors can directly determine the safety and feasibility of automated driving vehicles. Sensor calibration is the foundation block of any autonomous system and its constituent sensors and must be performed correctly before sensor fusion and obstacle detection processes may be implemented. This paper evaluates the capabilities and the technical performance of sensors which are commonly employed in autonomous vehicles, primarily focusing on a large selection of vision cameras, LiDAR sensors, and radar sensors and the various conditions in which such sensors may operate in practice. We present an overview of the three primary categories of sensor calibration and review existing open-source calibration packages for multi-sensor calibration and their compatibility with numerous commercial sensors. We also summarize the three main approaches to sensor fusion and review current state-of-the-art multi-sensor fusion techniques and algorithms for object detection in autonomous driving applications. The current paper, therefore, provides an end-to-end review of the hardware and software methods required for sensor fusion object detection. We conclude by highlighting some of the challenges in the sensor fusion field and propose possible future research directions for automated driving systems.

## 1. Introduction

According to the Global Status Report published by the World Health Organization (WHO), the reported number of annual road traffic deaths reached 1.35 million in 2018, making it the world’s eighth leading cause of unnatural death among people of all ages [1]. In the context of the European Union (EU), while there has been a decrease in the reported annual road fatalities, there is still more than 40,000 fatalities per annum, 90% of which were caused by human error. For this reason and to improve traffic flows, global investors have invested significantly to support the development of self-driving vehicles. Additionally, it is expected that the autonomous vehicles (AVs) will help to reduce the level of carbon emissions, and hence contribute to carbon emissions reduction targets [2].

AVs or self-driving vehicles provide the transportation capabilities of conventional vehicles but are largely capable of perceiving the environment and self-navigating with minimal or no human intervention. According to a report published by the Precedence Research, the global AV market size reached approximately 6500 units in 2019 and is predicted to experience a compound annual growth rate of 63.5% over the period 2020 to 2027 [3]. In 2009, Google secretly initiated its self-driving car project, currently known as Waymo (and presently a subsidiary of Google parent company Alphabet). In 2014, Waymo revealed a 100% autonomous car prototype without pedals and steering wheel [4]. To date, Waymo has achieved a significant milestone, whereby its AVs had collectively driven over 20 million miles on public roads in 25 cities in the United States of America (USA) [5]. Within the Irish context, in 2020, Jaguar Land Rover (JLR) Ireland has announced its collaboration with autonomous car hub in Shannon, Ireland, and will use 450 km of roads to test its next-generation AV technology [6].

**Figure 1:**
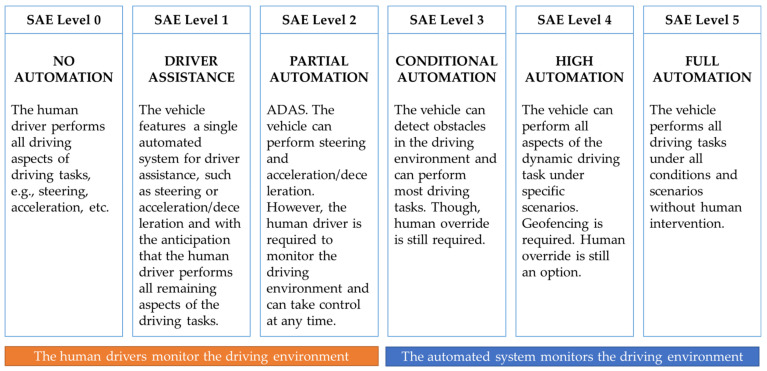
An overview of the six distinct levels of driving automation that were described in the Society of Automotive Engineers (SAE) J3016 standard. Readers interested in the comprehensive descriptions of each level are advised to refer to SAE International. Figure redrawn and modified based on depictions in [7].

In 2014, the SAE International, previously known as the Society of Automotive Engineers (SAE) introduced the J3016 “Levels of Driving Automation” standard for consumers. The J3016 standard defines the six distinct levels of driving automation, starting from SAE level 0 where the driver is in full control of the vehicle, to SAE level 5 where vehicles can control all aspects of the dynamic driving tasks without human intervention. The overview of these levels is depicted in Figure 1 and are often cited and referred to by industry in the safe design, development, testing, and deployment of highly automated vehicles (HAVs) [7]. Presently, automobile manufacturers such as Audi (Volkswagen) and Tesla adopted the SAE level 2 automation standards in developing its automation features, namely Tesla’s Autopilot [8] and Audi A8′s Traffic Jam Pilot [9,10]. Alphabet’s Waymo, on the other hand, has since 2016 evaluated a business model based on SAE level 4 self-driving taxi services that could generate fares within a limited area in Arizona, USA [11].

Most autonomous driving (AD) systems share many common challenges and limitations in real-world situations, e.g., safe driving and navigating in harsh weather conditions, and safe interactions with pedestrians and other vehicles. Harsh weather conditions, such as glare, snow, mist, rain, haze, and fog, can significantly impact the performance of the perception-based sensors for perception and navigation. Besides, the challenges for AD in adverse weather are faced in other constrained AD scenarios like agriculture and logistics. For on-road AVs, the complexity of these challenges increases because of the unexpected conditions and behaviors from other vehicles. For example, placing a yield sign in an intersection can change the behavior of the approaching vehicles. Hence, a comprehensive prediction module in AVs is critical to identify all position future motions to reduce collision hazards [12,13]. Although AD systems share many common challenges in real-world situations, they are differed noticeably in several aspects. For instance, unmanned tractors in agriculture farm navigates between crop rows in a fixed environment, while on-road vehicles must navigate through complex dynamic environment, such as crowds and traffics [14].

**Figure 2:**
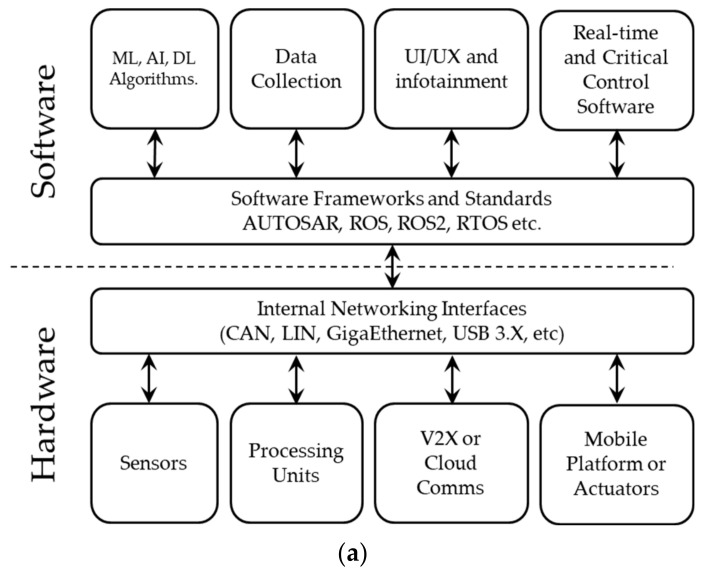

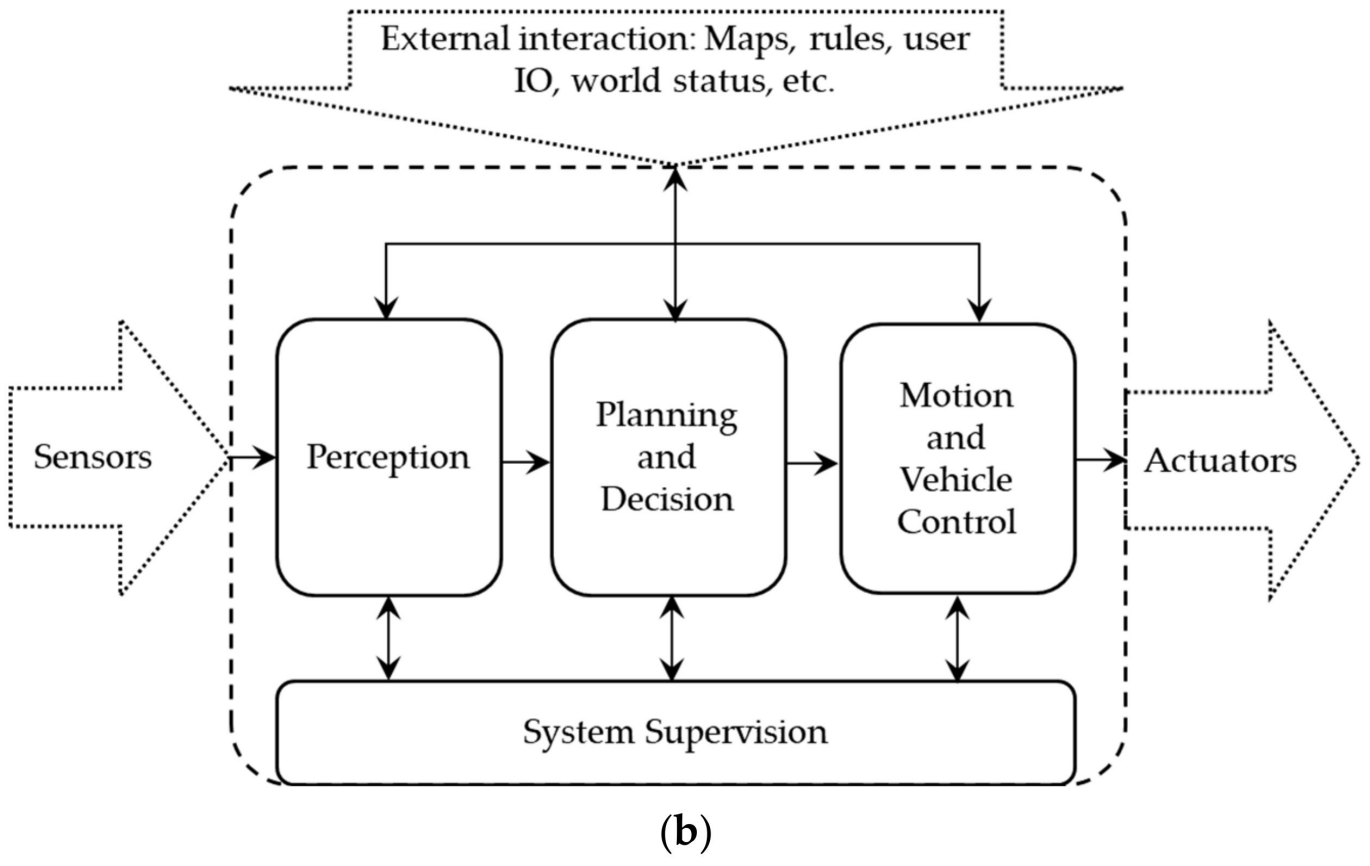
Architecture of an autonomous driving (AD) system from, (**a**) a technical perspective that describes the primary hardware and software components and their implementations; (**b**) a functional perspective that describes the four main functional blocks and the flow of information based on [15].

While AV systems may vary slightly from one to another, all are complex systems that consists of many subcomponents. In [15], the architecture of an AD system is introduced from a technical perspective, which incorporates the hardware and software components of the AD system, and from a functional perspective, which describes the processing blocks required within the AV, from data collection to the control of the vehicle. The hardware and software are the two primary layers from the technical perspective, and each layer includes various subcomponents that represent different aspects of the overall system. Some of the subcomponents serve as a backbone within its layer for communications between the hardware and software layers. In contrast, from the functional perspective, AV systems are composed of four primary functional blocks: *perception*, *planning and decision*, *motion and vehicle control*, and *system supervision*. These functional blocks are defined based on the processing stages and the flow of information from data collection to the control of the vehicle. The description of the technical and functional perspective of the architecture of an AV is represented in Figure 2. The detailed discussion of the AV architectures is beyond the scope of this paper (see [15] for a more detailed overview).

The sensing capabilities of an AV employing a diverse set of sensors is an essential element in the overall AD system; the cooperation and performance of these sensors can directly determine the viability and safety of an AV [16]. The selection of an appropriate array of sensors and their optimal configurations, which will, in essence be used to imitate the human ability to perceive and formulate a reliable picture of the environment, is one of the primary considerations in any AD system.

It is always essential to consider the advantages, disadvantages, and limitations of the selected group of sensors, i.e., *smart sensors* and *non-smart sensors*. The definition of “smart sensor” has evolved over the past decades along with the emergence of the Internet of Things (IoT), a system of interrelated, internet-connected objects (devices) that can collect and transfer data over the wireless network without human intervention. In the IoT context, a smart sensor is a device that can condition the input signals, process, and interpret the data, and make decisions without a separate computer [17]. In addition, in the AV context, range sensors for environment perception, e.g., cameras, LiDARs, and radars, may be considered “smart” when the sensors provide for example, target tracking, event descriptions, and other information, as part of their output. In contrast, a “non-smart” sensor is a device that only conditions the sensor raw data or waveforms and transfers the data for remote processing. It requires external computing resources to process and interpret the data to provide additional information about the environment. Ultimately, a sensor is only considered “smart” when the computer resources is an integral part of the physical sensor design [18]. Invariably, the overall performance of an AV system is greatly enhanced with multiple sensors of different types (smart/non-smart) and modalities (visual, infrared and radio waves) operating at different range and bandwidth (data rate) and with the data of each being incorporated to produce a fused output [17,18,19]. Multi-sensor fusion is effectively now a requisite process in all AD systems to overcome the shortcomings of individual sensor types, improving the efficiency and reliability of the overall AD system.

Several reviews have been published recently on the topic of multi-sensor fusion, some of them describing the architectural structure and sensor technologies in AVs [15,20,21], or focusing on the processing stages like sensor calibration, state estimation, object and tracking [22,23,24], or detailing techniques used for multi-sensor fusion, like deep learning-based approaches [19,25,26]. Table 1 below summarizes some of the recent studies in sensor and sensor fusion technologies in AD systems.

The present review paper will extend across the three major considerations in sensor fusion for AVs: Firstly, operating principles and characteristics of sensor modalities, including a comparison of commercially available hardware; secondly, the three aspects of sensor calibration, the main open-source calibration systems and their compatibility with commercial sensors; and thirdly, on sensor fusion methods and algorithms for obstacle detection in AV environments. Section 2 provides an overview of the existing sensing modalities used in AVs, primarily focusing on cameras, LiDARs, and radars, including their advantages and disadvantages, and limitations in different environmental conditions. Section 3 discusses the necessity of sensor calibration in AVs, an overview of an existing calibration package which addresses all the main aspects required by any calibration system, followed by the current developments of sensor fusion approaches for obstacle detection and its challenges for safe and reliable environment perception. Section 4 presents a summary review and recommendations for future research in AVs.

## 2. Sensor Technology in Autonomous Vehicles

Sensors are devices that map the detected events or changes in the surroundings to a quantitative measurement for further processing. In general, sensors are classified into two classes based on their operational principal. Proprioceptive sensors, or internal state sensors, capture the dynamical state and measures the internal values of a dynamic system, e.g., force, angular rate, wheel load, battery voltage, et cetera. Examples of the proprioceptive sensors include Inertia Measurement Units (IMU), encoders, inertial sensors (gyroscopes and magnetometers), and positioning sensors (Global Navigation Satellite System (GNSS) receivers). In contrast, the exteroceptive sensors, or external state sensors, sense and acquire information such as distance measurements or light intensity from the surroundings of the system. Cameras, Radio Detection and Ranging (Radar), Light Detection and Ranging (LiDAR), and ultrasonic sensors are examples of the exteroceptive sensors. Additionally, sensors can either be passive sensors or active sensors. Passive sensors receive energy emitting from the surroundings to produce outputs, e.g., vision cameras. Conversely, active sensors emit energy into the environment and measure the environmental “reaction” to that energy to produce outputs, such as with LiDAR and radar sensors [27,28,29].

**Figure 3:**
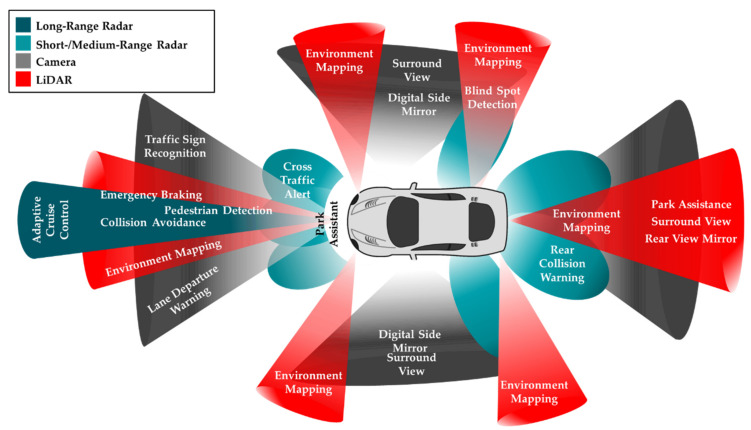
An example of the type and positioning of sensors in an automated vehicle to enable the vehicles perception of its surrounding. Red areas indicate the LiDAR coverage, grey areas show the camera coverage around the vehicle, blue areas display the coverage of short-range and medium-range radars, and green areas indicate the coverage of long-range radar, along with the applications the sensors enable—as depicted in [32] (redrawn).

In AVs, sensors are critical to the perception of the surroundings and localization of the vehicles for path planning and decision making, essential precursors for controlling the motion of the vehicle. AV primarily utilizes multiple vision cameras, radar sensors, LiDAR sensors, and ultrasonic sensors to perceive its environment. Additionally, other sensors, including the Global Navigation Satellite System (GNSS), IMU, and vehicle odometry sensors are used to determine the relative and absolute positions of the vehicle [30]. The relative localization of an AV refers to the vehicles referencing of its coordinates in relation to the surrounding landmarks, while absolute localization refers to the vehicle referencing its position in relation to a global reference frame (world) [31]. The placement of sensors for environment perception on typical AV applications, their coverage, and applications are shown in Figure 3. The reader will appreciate that in a moving vehicle, there is a more complete coverage of the vehicle’s surroundings. The individual and relative positioning of multiple sensors are critical for precise and accurate object detection and therefore reliably and safely performing any subsequent actions [32]. In general, it is challenging to generate adequate information from a single independent source in AD. This section reviews the advantages and shortcomings of the three primary sensors: cameras, LiDARs and radars, for environment perception in AV applications.

### 2.1. Camera

Cameras are one of the most adopted technology for perceiving the surroundings. A camera works on the principle of detecting lights emitted from the surroundings on a photosensitive surface (image plane) through a camera lens (mounted in front of the sensor) to produce clear images of the surrounding [20,30]. Cameras are relatively inexpensive and with appropriate software, can detect both moving and static obstacles within their field of view and provides high-resolution images of the surroundings. These capabilities allow the perception system of the vehicle to identify road signs, traffic lights, road lane markings and barriers in the case of road traffic vehicles and a host of other articles in the case of off-road vehicles. The camera system in an AV may employ monocular cameras or binocular cameras, or a combination of both. As the name implies, the monocular camera system utilizes a single camera to create a series of images. The conventional RGB monocular cameras are fundamentally more limited than stereo cameras in that they lack native depth information, although in some applications or more advanced monocular cameras using the dual-pixel autofocus hardware, depth information may be calculated using complex algorithms [33,34,35]. As a result, two cameras are often installed side-by-side to form a binocular came-ra system in autonomous vehicles.

The stereo camera, also known as a binocular camera, imitates the perception of depth found in animals, whereby the “disparity” between the slightly different images formed in each eye is (subconsciously) employed to provide a sense of depth. Stereo cameras contain two image sensors, separated by a baseline. The term baseline refers to the distance between the two image sensors (and is generally cited in the specifications of stereo cameras), and it differs depending on the camera’s model. For example, the Orbbec 3D cameras reviewed in [36] for Autonomous Intelligent Vehicles (AIV) has a baseline of 75 mm for both the Persee and Astra series cameras [37]. As in the case of animal vision, the disparity maps calculated from the stereo camera imagery permit the generation of depth maps using epipolar geometry and triangulation methods (detailed discussion of the disparity calculations algorithms is beyond the scope of this paper). Reference [38] uses the “stereo_image_proc” modules in Robotic Operating System (ROS), an open source, meta-operating system for robotics [39], to perform stereo vision processing before implementing SLAM (simultaneous localization and mapping) and autonomous navigation. Table 2 shows the general specifications for binocular cameras from different manufacturers.

Other commonly employed cameras in AVs for perception of the surroundings include fisheye cameras [52,53,54]. Fisheye cameras are commonly employed in near-field sensing applications, such as parking and traffic jam assistance, and require only four cameras to provide a 360-degree view of the surroundings. Reference [52] proposed a fisheye surround-view system and the convolutional neural network (CNN) architecture for moving object segmentation in an autonomous driving environment, running at 15 frames per second at an accuracy of 40% Intersection over Union (IoU, in approximate terms, an evaluation metric that calculates the area of overlap between the target mask (ground truth) and predicted mask), and 69.5% mean IoU.

The deviation in lens geometry from the ideal/nominal geometry will result in image distortion, such that in extreme cases, e.g., ultra-wide lenses employed in fisheye cameras, straight lines in the physical scene may become curvilinear. In photography, the deviations in camera lens geometry are generally referred to as optical distortion, and are commonly categorized as pincushion distortion, barrel distortion, and moustache distortion. Such distortions may introduce an error in the estimated location of the detected obstacles or features in the image. Hence, it is often a require to “intrinsically calibrate” the camera to estimate the camera parameters and rectify the geometric distortions [55]. We present a detailed discussion of the camera intrinsic calibration and the commonly employed method in Section 3.1.1. Further, it is known that the quality (resolution) of images captured by the cameras may significantly affected by lighting and adverse weather conditions, e.g., snow, intense sun glare, rainstorm, hazy weather, et cetera. Other disadvantages of cameras may include the requirement for large computation power while analyzing the image data [20].

Given the above, cameras are a ubiquitous technology that provides high-resolution videos and images, including color and texture information of the perceived surroundings. Common uses of the camera data on AVs include traffic signs recognition, traffic lights recognition, and road lane marking detection. As the camera’s performance and the creation of high-fidelity images are highly dependent on the environmental conditions and illumination, image data are often fused with other sensor data such as radar and LiDAR data, to generate reliable and accurate environment perception in AD.

### 2.2. LiDAR

Light Detection and Ranging, or LiDAR, was first established in the 1960s and was widely used in the mapping of aeronautical and aerospace terrain. In the mid-1990s, laser scanners manufacturers produced and delivered the first commercial LiDARs with 2000 to 25,000 pulses per second (PPS) for topographic mapping applications [56]. The development of LiDAR technologies has evolved continuously at a significant pace over the past few decades and is currently one of the cores perception technologies for Advanced Driver Assistance System (ADAS) and AD vehicles. LiDAR is a remote sensing technology that operates on principle of emitting pulses of infrared beams or laser light which reflect off target objects. These reflections are detected by the instrument and the interval taken between emission and receiving of the light pulse enables the estimation of distance. As the LiDAR scans its surroundings, it generates a 3D representation of the scene in the form of a point cloud [20].

The rapid growth of research and commercial enterprises relating to autonomous robots, drones, humanoid robots, and AVs has established a high demand for LiDAR sensors due to its performance attributes such as measurement range and accuracy, robustness to surrounding changes and high scanning speed (or refresh rate)—for example, typical instruments in use today may register up to 200,000 points per second or more, covering 360° rotation and a vertical field of view of 30°. As a result, many LiDAR sensor companies have emerged and have been introducing new technologies to address these demands in recent years. Hence, the revenue of the automotive LiDAR market is forecasted to reach a total of 6910 million USD by 2025 [57]. The wavelengths of the current state-of-the-art LiDAR sensors exploited in AVs are commonly 905 nm (nanometers)—safest types of lasers (Class 1), which suffers lower absorption water than for example 1550 nm wavelength sensors which were previously employed [58]. A study in reference [59] found that the 905 nm systems can provide higher resolution of point clouds in adverse weather conditions like fog and rains. The 905 nm LiDAR systems, however, are still partly sensitive to fog and precipitation: a recent study in [60] conveyed that harsh weather conditions like fogs and snows could degrade the performance of the sensor by 25%.

The three primary variants of LiDAR sensors that can be applied in a wide range of applications include 1D, 2D and 3D LiDAR. LiDAR sensors output data as a series of points, also known as point cloud data (PCD) in either 1D, 2D and 3D spaces and the intensity information of the objects. For 3D LiDAR sensors, the PCD contains the *x*, *y*, *z* coordinates and the intensity information of the obstacles within the scene or surroundings. For AD applications, LiDAR sensors with 64- or 128- channels are commonly employed to generate laser images (or point cloud data) in high resolution [61,62].

- 1D or one-dimensional sensors measure only the distance information (x-coordinates) of objects in the surroundings.
- 2D or two-dimensional sensors provides additional information about the angle (y-coordinates) of the targeted objects.
- 3D or three-dimensional sensors fire laser beams across the vertical axes to measure the elevation (z-coordinates) of objects around the surroundings.

LiDAR sensors can further be categorized as mechanical LiDAR or solid-state LiDAR (SSL). The mechanical LiDAR is the most popular long-range environment scanning solution in the field of AV research and development. It uses the high-grade optics and rotary lenses driven by an electric motor to direct the laser beams and capture the desired field of view (FoV) around the AV. The rotating lenses can achieve a 360° horizontal FoV covering the vehicle surroundings. Contrarily, the SSLs eliminate the use of rotating lenses and thus avoiding mechanical failure. SSLs use a multiplicity of micro-structured waveguides to direct the laser beams to perceive the surroundings. These LiDARs have gained interest in recent years as an alternative to the spinning LiDARs due to their robustness, reliability, and generally lower costs than the mechanical counterparts. However, they have a smaller and limited horizontal FoV, typically 120° or less, than the traditional mechanical LiDARs [30,63].

Reference [64] compares and analyzes 12 spinning LiDAR sensors that are currently available in the market from various LiDAR manufacturers. In [64], different models and laser configurations are evaluated in three different scenarios and environments, including dynamic traffic, adverse weather generated in a weather simulation chamber, and static targets. The results demonstrated that the Ouster OS1-16 LiDAR model had the lowest average number of points on reflective targets and the performance of spinning LiDARs are strongly affected by intense illumination and adverse weather, notable where precipitation is high and there is non-uniform or heavy fog. Table 3 shows the general specifications of each tested LiDAR sensor in the study of [64] (comprehensive device specifications are presented as well in [65]). In addition, we extended the summarized general specifications in the study of [64,65] with other LiDARs, including Hokuyo 210° spinning LiDAR and SSLs from Cepton, SICK, and IBEO, and the commonly used ROS drivers for data acquisition from our initial findings.

Laser returns are discrete observations that are recorded when a laser pulse is intercepted and reflected by the targets. LiDARs can collect multiple returns from the same laser pulse and modern sensors can record up to five returns from each laser pulse. For instance, the Velodyne VLP-32C LiDAR analyze multiple returns and reports either the strongest, last, or dual return, depending on the laser return mode configurations. In single laser return mode (strongest return or last return), the sensor analyzes lights received from the laser beam in one direction to determine the distance and intensity information and subsequently employs this information to determine the last return or strongest return. In contrast, sensors in dual return configuration mode will return both the strongest and last return measurements. However, the second-strongest measurements will return as the strongest if the strongest return measurements are like the last return measurements. Not to mention that points with insufficient intensity will be disregarded [66].

In general, at present, 3D spinning LiDARs are more commonly applied in self-driving vehicles to provide a reliable and precise perception of in day and night due to its broader field of view, farther detection range and depth perception. The acquired data in point cloud format provides a dense 3D spatial representation (or “laser image”) of the AVs’ surroundings. LiDAR sensors do not provide color information of the surroundings compared to the camera systems and this is one reason that the PCD is often fused with data from different sensors using sensor fusion algorithms.

**Table 3:** General specifications of the tested LiDARs from [64,65] and other LiDARs that were reviewed in the current work. The acronyms from left to right (first row) are frames per second (FPS); accuracy (Acc.); detection range (RNG); vertical FoV (VFOV); horizontal FoV (HFOV); horizontal resolution (HR); vertical resolution (VR); wavelength (*λ*); diameter (Ø); sensor drivers for Robotic Operating System (ROS Drv.); and reference for further information (Ref.). The “-” symbol in table below indicates that the specifications were not mentioned in product datasheet.

	Company	Model	Channels or Layers	FPS (Hz)	Acc. (m)	RNG (m)	VFOV (°)	HFOV (°)	HR (°)	VR (°)	*λ* (nm)	Ø (mm)	ROS Drv.	Ref.
**Mechanical/Spinning LiDARs**	**Velodyne**	VLP-16	16	5–20	±0.03	1…100	30	360	0.1–0.4	2	903	103.3	[67]	[51,68,69,70]
		VLP-32C	32	5–20	±0.03	1…200	40	360	0.1–0.4	0.33 ^1^	903	103		
		HDL-32E	32	5–20	±0.02	2…100	41.33	360	0.08–0.33	1.33	903	85.3		
		HDL-64E	64	5–20	±0.02	3…120	26.8	360	0.09	0.33	903	223.5		
		VLS-128 Alpha Prime	128	5–20	±0.03	max 245	40	360	0.1–0.4	0.11 ^1^	903	165.5	-	
	**Hesai**	Pandar64	64	10,20	±0.02	0.3…200	40	360	0.2,0.4	0.167 ^1^	905	116	[71]	[72]
		Pandar40P	40	10,20	±0.02	0.3…200	40	360	0.2,0.4	0.167 ^1^	905	116		[73]
	**Ouster**	OS1–64 Gen 1	64	10,20	±0.03	0.8…120	33.2	360	0.7,0.35, 0.17	0.53	850	85	[74]	[75,76]
		OS1-16 Gen 1	16	10,20	±0.03	0.8…120	33.2	360		0.53	850	85		
	**RoboSense**	RS-Lidar32	32	5,10,20	±0.03	0.4…200	40	360	0.1–0.4	0.33 ^1^	905	114	[77]	[78]
	**LeiShen**	C32-151A	32	5,10,20	±0.02	0.5…70	32	360	0.09, 0.18,0.36	1	905	120	[79]	[80]
		C16-700B	16	5,10,20	±0.02	0.5…150	30	360		2	905	102	[81]	[82]
	**Hokuyo**	YVT-35LX-F0	-	20 ^3^	±0.05 ^3^	0.3…35 ^3^	40	210	-	-	905	^◊^	[83]	[84]
**Solid State LiDARs**	**IBEO**	LUX 4L Standard	4	25	0.1	50 ^2^	3.2	110	0.25	0.8	905	^◊^	[85]	[86]
		LUX HD	4	25	0.1	50 ^2^	3.2	110	0.25	0.8	905	^◊^		[87]
		LUX 8L	8	25	0.1	30 ^2^	6.4	110	0.25	0.8	905	^◊^		[88]
	**SICK**	LD-MRS400102S01 HD	4	50	-	30 ^2^	3.2	110	0.125…0.5		-	^◊^	[85]	[89]
		LD-MRS800001S01	8	50	-	50 ^2^	6.4	110	0.125…0.5		-	^◊^		[90]
	**Cepton**	Vista P60	-	10	-	200	22	60	0.25	0.25	905	^◊^	[91]	[92]
		Vista P90	-	10	-	200	27	90	0.25	0.25	905	^◊^		[93]
		Vista X90	-	40	-	200	25	90	0.13	0.13	905	^◊^		[94]

^1^ Stated resolution refer to the minimum (or finest) resolutions, as these sensors have variable angle difference between central and more apical/basal beams. ^2^ The documented maximum detection range is at 10% remission rate (or reflectivity rate, is a measurement of diffuse reflection on surfaces). ^3^ The indicated FPS refers to the sensor’s non-interlace mode. The detection range and accuracy stated refer to white paper detections below 15m at center of vertical scan. ^◊^ Dimension/Size of the sensors are in rectangular shape: width (W) × height (H) × depth (D)—see individual references for actual dimensions.

### 2.3. Radar

Radio Detection and Ranging, or Radar, was first established before World War II and operated on the principle of radiating electromagnetic (EM) waves within the area of interest and receiving the scattered waves (or reflections) of targets for further signal processing and establishing range information about the targets. It uses the Doppler property of EM waves to determine the relative speed and relative position of the detected obstacles [30], The Doppler effect, also known as Doppler shift, refers to the variations or shifts in wave frequency arising from relative motion between a wave source and its targets. For instance, the frequency of the received signal increases (shorter waves) when the target moves towards the direction of the radar system [95]. The general mathematical equation of Doppler frequency shift of a radar can be represented as [96,97]:

(1) $$f_{D}\;=\;\frac{2\;\times \;V_{r\;}\times f}{C}=\frac{2\;\times \;V_{r}}{\lambda }$$

where $f_{D}$ is the Doppler frequency in Hertz (Hz); $V_{r}$ is the relative speed of the target; *f* is the frequency of the transmitted signal; *C* is the speed of light (3 × 10^8^ m/s) and *λ* is the wavelength of the emitted energy. In practice, the Doppler frequency change in a radar occurs twice; firstly, when the EM waves are emitted to the target and secondly, during the reflection of the Doppler shifted energy to the radar (source).

**Figure 4:**
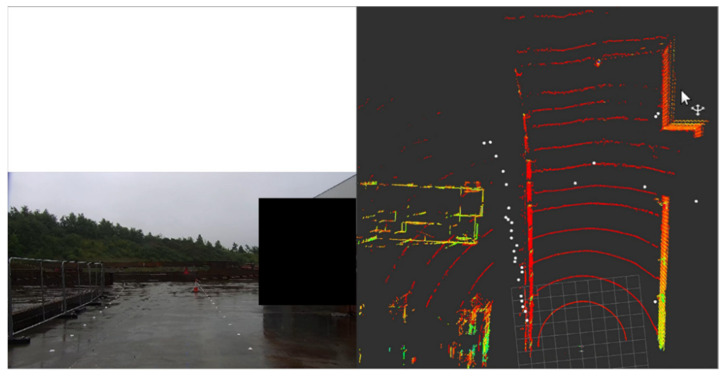
Visualization (before correction for several degrees of sensor misalignment) of false-positive detections in current exploratory research. The colored points in the point clouds visualization represent LiDAR point cloud data and white points represent radar point cloud data. Several false-positive radar detections are highlighted by the grey rectangle, located at approximately 5–7 m from the radar sensor. The radar sensor in present setup is in short-range mode (maximum detection range is 19 m); hence, the traffic cone located at 20 m is not detectable.

Commercial radars available on the market currently operate at 24 GHz (Gigahertz), 60 GHz, 77 GHz, and 79 GHz frequencies. Compared to the 79 GHz radar sensors, 24 GHz radar sensors have a more limited resolution of range, velocity, and angle, leading to problems in identifying and reacting to multiple hazards and are predicted to be phased out in the future [30]. The propagation of the EM waves (radar) is impervious to adverse weather conditions and radar function is independent of the environment illumination conditions; hence, they can operate at day or night in foggy, snowy, or cloudy conditions. Among the drawbacks of radar sensors are the false detection of metal objects around the perceived surroundings like road signs or guardrails and the challenges of distinguishing static, stationary objects [21]. For instance, the difference between an animal carcass (static objects) and the road may pose a challenge for radars to resolve due to the similarity in Doppler shift [98]. Initial findings within the present research using 79 GHz automotive radar sensor (SmartMicro [22]) demonstrated in [22] showed a high frequency of false-positive detections within the area of interest. Figure 4 shows an example of the false-positive detections of objects at about 5–7 m from the mounted sensors.

Radar sensors in AD vehicles are commonly integrated invisibly in several locations, such as on the roof near the top of the windshield, behind the vehicle bumpers or brand emblems. It is essential to ensure the precision of mounting positions and orientations of radars in production, as any angular misalignment could have fatal consequences for operation of the vehicle, such errors including false or late detections of obstacles around the surroundings [99,100]. Medium-Range Radar (MRR), Long-Range Radar (LRR), and Short-Range Radar (SRR) are the three major categories of automotive radar systems. AV manufacturers utilize SRR for packing assistance and collision proximity warning, MRR for side/rear collision avoidance system and blind-spot detection and LRR for adaptive cruise control and early detection applications [30]. We reviewed the general specifications of several radar sensors from various manufacturers, such as SmartMicro, Continental, and Aptiv Delphi and an overview is presented in Table 4.

In general, radar sensors are one of the well-known sensors in the autonomous systems and are commonly employed in AVs to provide a reliable and precise perception of obstacles in day and night because of its capability to function irrespective of illumination and adverse weather conditions. It provides additional information, such as speed of the detected moving obstacles and can perform mapping in either short, medium, or long-range depending on the configuration mode. The radar sensor, however, is not generally suitable for object recognition applications because of their coarse resolutions compared to cameras. Therefore, AV researchers often fuse radar information with other sensory data, such as camera and LiDAR, to compensate for the limitations of radar sensors.

## 3. Sensor Calibration and Sensor Fusion for Object Detection

**Figure 5:**
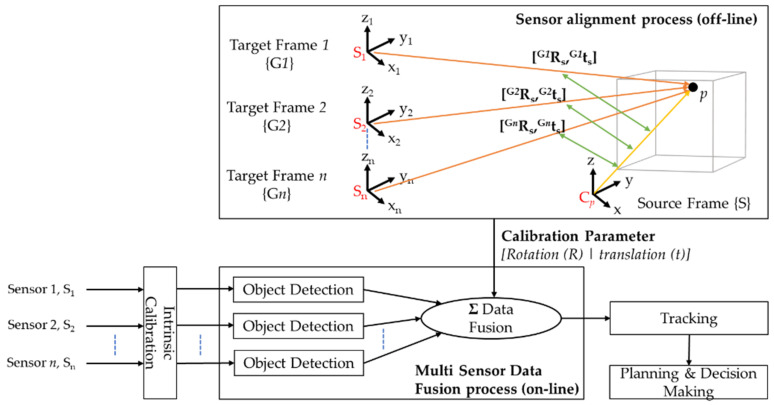
The structure of Multi-Sensor Data Fusion (MSDF) framework for *n* given sensors. It consists of a sensor alignment process (estimation of calibration parameters—rotation matrix and translations vector) and an object detection process which contains *n* processing chains, each provides a list of the detected obstacles. Figure redrawn based on depictions in [118], but with the inclusion of an intrinsic calibration process.

According to an article from Lyft Level 5, a self-driving division of Lyft in the United States [117], sensor calibration is one of the least discussed topics in the development of autonomous systems. It is the foundation block of an autonomous system and their constituent sensors, and it is a requisite processing step before implementing sensor fusion techniques and algorithms for AD applications. Sensor calibration notifies the autonomous system about the sensors’ position and orientation in real-world coordinates by comparing the relative positions of known features as detected by the sensors. Precise calibrations are vital for further processing steps, such as sensor fusion and implementation of algorithms for obstacle detection, localization and mapping, and control. Further, sensor fusion is one of the essential tasks in AD applications that fuses information obtained from multiple sensors to reduce the uncertainties compared to when sensors are used individually. The fusion algorithms are used principally in the perception block of the overall AD architecture, which involves the object detection sub-processes. Reference [118] presented the Multi-Sensor Data Fusion (MSDF) framework for AV perception tasks, as depicted in Figure 5. The MSDF framework consists of a sensor alignment process and several object detection processing chains, and subsequently integrates the outputs from sensor alignment and object detection for further processing tasks.

Section 3.1 highlights the three categories of calibrations: intrinsic calibration, extrinsic calibration, and temporal calibration [119] and provides an overview of an existing calibration packages which has been employed in the current research. Section 3.2 reviews the three sensor approaches, namely high-level fusion (HLF), low-level fusion (LLF), and mid-level fusion (MLF) for object detection and summarizes the commonly employed algorithms, followed by the challenges of sensor fusion for safe and reliable environment perception.

### 3.1. Sensor Calibrations

#### 3.1.1. Intrinsic Calibration Overview

Intrinsic calibration addresses sensor-specific parameters and is conducted before implementing extrinsic calibration and obstacle detection algorithms. Intrinsic calibration estimates the internal or intrinsic parameters of a sensor, e.g., focal lengths of a vision camera, which correct for systematic or deterministic aberrations (errors). These parameters are anticipated to be consistent once the intrinsic parameters are estimated [120]. It is known through personal communication that Velodyne LiDARs are calibrated to 10% reflectivity of the National Institute of Standards and Technology (NIST) targets. Therefore, the reflectance of the obstacles below the 10% reflectivity rate may not be detected by the LiDAR [121]. Algorithms and methods for intrinsic calibration of sensors have received considerable attention with significant advancement over the last number of years and now, are well-established in the literature. These algorithms and methodologies may vary from one sensor to another [122,123,124,125,126,127,128,129]. This subsection aims to provide an overview of the most used calibration targets and the calibration methodologies for the pinhole camera model.

**Figure 6:**
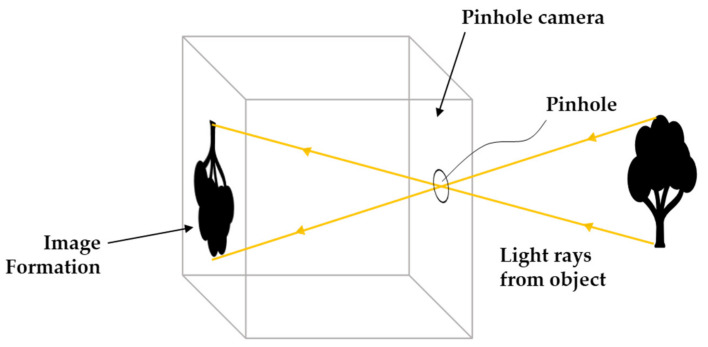
A graphical representation of the pinhole camera. The pinhole (aperture) restraints the light rays from the target from entering the pinhole; hence, affecting the brightness of the captured image (during image formation). A large pinhole (a wide opening) will result in a brighter image but is less clear due to blurriness on both background and foreground. Figure redrawn based on depictions in [132,133].

The pinhole camera model is a well-known and commonly used model (inspired by the simplest cameras [130]) in computer vision applications, which describes the mathematical relationship of the projection of points in 3D space on to a 2D image plane [131]. Figure 6 visualizes the camera pinhole model, which consists of a closed box with a small opening (pinhole) on the front side through which the light rays from a target enters and produces an image on the opposing camera wall (image plane) [132].

**Figure 7:**
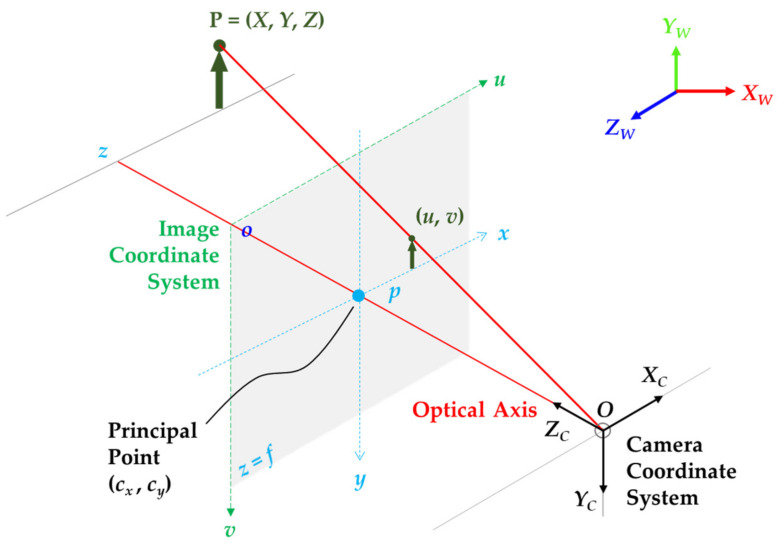
The pinhole camera model from a mathematical perspective. The optical axis (also referred to as principal axis) aligns with the *Z*-axis of the camera coordinate system (*Z_C_*), and the intersections between the image plane and the optical axis is referred to as the principal points (*c_x_*, *c_y_*). The pinhole opening serves as the origin (*O*) of the camera coordinate system (*X_C_*, *Y_C_*, *Z_C_*) and the distance between the pinhole and the image plane is referred to as the focal length (*f*). Computer vision convention uses right-handed system with the *z*-axis pointing toward the target from the direction of the pinhole opening, while *y*-axis pointing downward, and *x*-axis rightward. Conventionally, from a viewer’s perspective, the origin (*o*) of the 2D image coordinate system (*x*, *y*) is at the top-left corner of the image plane with *x*-axis pointing rightward, and *y*-axis downward. The (*u*, *v*) coordinates on the image plane refers to the projection of points in pixels. Figure redrawn based on depictions in [125,134,135].

From a mathematical perspective (Figure 7), the model involves a 3D camera coordinate system and a 2D image coordinate system to calibrate the camera using a perspective transformation method [134,135]. The calibration process involves utilizing the *extrinsic* parameters (a 3 × 4 matrix that consists of the rotation and translation [R | t] transformation) to transform the 3D points in world coordinate space (*X_W_*, *Y_W_*, *Z_W_*) into their corresponding 3D camera coordinates (*X_C_*, *Y_C_*, *Z_C_*). In addition, it involves employing the *intrinsic* parameters (also referred to as the 3 × 3 intrinsic matrix, *K* [136]), to transform the 3D camera coordinates into the 2D image coordinates (*x*, *y*).

The perspective transformation method outputs a 4 × 3 camera matrix (*P*), also referred to as the projection matrix, which consists of the intrinsic and extrinsic parameters to transform 3D world coordinate space into the 2D image coordinates. It should be stressed that the extrinsic calibration parameters in the camera calibration context differ from the extrinsic calibration process of one or more sensors relative to another sensor. It is known that the camera matrix does not account for any lens distortion—the ideal pinhole camera lacking a lens. The general mathematical equation of the perspective method is represented as [125,134,137,138]:

(2) $$P=K\;\left[R\;\left|\;t\right.\right]\;\mathrm{or}\;P=\left[\begin{array}{ccc} f_{x} & s & c_{x}\\ 0 & f_{y} & c_{y}\\ 0 & 0 & 1 \end{array}\right]\left[\begin{array}{cccc} r_{11} & r_{12} & r_{13} & t_{1}\\ r_{21} & r_{22} & r_{23} & t_{2}\\ r_{31} & r_{32} & r_{33} & t_{3} \end{array}\right]\left[\begin{array}{c} X_{w}\\ Y_{w}\\ Z_{w}\\ 1 \end{array}\right]$$

where *P* is the 4 × 3 camera matrix; [R|t] represents the extrinsic parameters (rotation and translation) to transform the 3D world points (*X_W_*, *Y_W_*, *Z_W_*) into camera coordinates; and *K* is the intrinsic matrix of the pinhole camera that consists of the geometry properties of a camera, such as axis skew (*s*), optical centers or principal points offset (*c_x_*, *c_y_*) and focal lengths (*f_x_*, *f_y_*). The focal length (*f*) of a camera refers to the distance between the pinhole and the image plane and it determines the projection scale of an image. Hence, a smaller focal length will result in a smaller image and a larger viewing angle [132]. A detailed discussion of the projection of 3D world points into a 2D image plane, estimation of camera lens distortion, and the implementations are beyond the scope of this paper (see [132,133] for a more comprehensive overview).

Camera calibration (or camera re-sectioning [137]) is the process of determining the intrinsic and extrinsic parameters that comprise the camera matrix. Camera calibration is one of the quintessential issues in computer vision and photogrammetry and has received considerable attention over the last number of years. A variety of calibration techniques, [124,125,126,133,139,140,141,142] to cite a few, have been developed to accommodate various applications, such as AVs, Unmanned Surface Vehicle (USV) or underwater 3D reconstructions. Reference [141] classified these techniques into:

- Photogrammetric calibration. This approach uses the known calibration points observed from a calibration object (usually a planar pattern) where the geometry in the 3D world space is known with high precision.
- Self-calibration. This approach utilizes the correspondence between the captured images from a moving camera in a static scene to estimate the camera intrinsic and extrinsic parameters.

**Figure 8:**
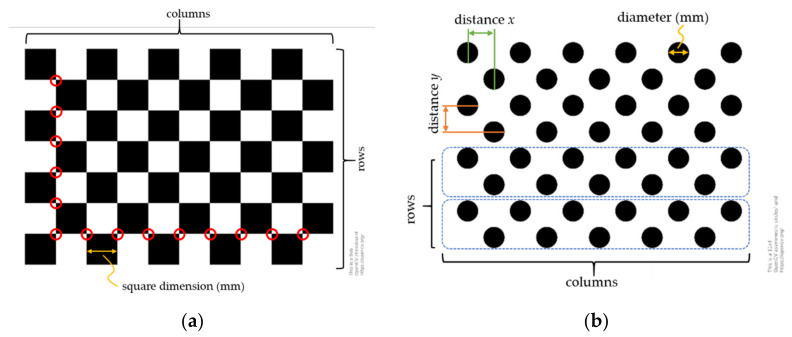
The most employed patterns for camera calibration. (**a**) A 7 rows × 10 columns checkerboard pattern. The calibration uses the interior vertex points of the checkerboard pattern; thus, the checkerboard in (**a**) will utilize the 6 × 9 interior vertex points (some of which are circled in red) during calibration. (**b**) A 4 rows × 11 columns asymmetrical circular grid pattern. The calibration uses the information from circles (or “blobs” in image processing terms) detection to calibrate the camera. Other planar patterns include symmetrical circular grid and ChArUco patterns (a combination of checkerboard pattern and ArUco pattern) [128,137,141]. Figures source from OpenCV and modified.

The well-known *Zhang method* is one of the most used camera calibration techniques. It uses a combination of photogrammetric calibration and self-calibration techniques to estimate the camera matrix. It uses the known calibration points observed from a planar pattern (Figure 8) from multiple orientations (at least two) and the correspondence between the calibration points in various positions to estimate the camera matrix. In addition, the Zhang method for camera calibration does not require the motion information when either the camera or the planar pattern are moved relative to each other [141].

The popular open source “camera_calibration” package in ROS offers several pre-implemented scripts to calibrate monocular, stereo, and fisheye cameras using the planar pattern as a calibration target. The calibration result includes the intrinsic matrix of a distorted image, distortion parameters, rectification matrix (stereo cameras only), camera matrix or projection matrix, and other operational parameters such as binning and region of interest (ROI). The calibration package was built based on the OpenCV camera calibration and 3D reconstruction package. Further, the calibration algorithm was implemented based on the well-known Zhang method and the camera calibration toolbox for MATLAB by Bouguet, J.Y. [128,134].

In general, camera calibration results are no longer applicable if the camera’s zoom (focal length) has changed. It should be noted that in our experience, radar and LiDAR sensors are factory intrinsic-calibrated.

#### 3.1.2. Extrinsic Calibration Overview

Extrinsic calibration is a rigid transformation (or Euclidean transformation) that maps the points from one 3D coordinate system to another, for example, a rigid transformation of points from the 3D world or 3D LiDAR coordinate system to the 3D camera coordinate system. The extrinsic calibration estimates the position and orientation of the sensor relative to the three orthogonal axes of 3D space (also known as the 6 *degrees of freedoms*, 6DoF) with respect to an external frame of reference [119,143]. The calibration process outputs the extrinsic parameters that consist of the rotation (*R*) and translation (*t*) information of the sensor and is commonly represented in a 3 × 4 matrix, as shown in Equation (2). This section aims to provide a comparative overview of existing open-source multi-sensor extrinsic calibration packages and a summary of algorithms proposed in the literature for extrinsic calibration of camera, LiDAR, and radar sensors comprising a sensor fusion system.

The studies of extrinsic calibration and the methodologies are well-established in the literature, see reference [143,144,145,146,147,148,149,150,151] for example. Though, the extrinsic calibration of multiple sensors with various physical measurement principles can pose a challenge in multi-sensor systems. For instance, it is often challenging to match the corresponding features between camera images (dense data in pixels) and 3D LiDAR or radar point clouds (sparse depth data without color information) [144]. The *target-based* extrinsic calibration approach employs specially designed calibration targets, such as marker-less planar pattern [51], checkerboard pattern [145], orthogonal and trihedral reflector [51,143,146,148], circular pattern to calibrate multiple sensor modalities in autonomous systems. The *targetless* extrinsic calibration approach leverages the estimated motion by individual sensors or utilizes the features in the perceiving environment to calibrate the sensors. However, employing the perceived environment features requires multimodal sensors to extract the same features within the environment and is sensitive to the calibration environment [144,149].

A comparative overview of existing extrinsic calibration tools in [146] reported that the available tools only addressed pairwise calibrations of a maximum of two sensing modalities. For instance, the framework presented in [143] uses a *coarse to fine extrinsic calibration* approach to calibrate the RGB camera with a Velodyne LiDAR. The algorithm utilizes a novel 3D marker with four circular holes to estimate the coarse calibration parameters and further refine these parameters using the dense search approach to estimate a more accurate calibration in the small 6DoF calibration parameters subspace. Reference [150] presented an extrinsic calibration algorithm which utilizes the *Planar Surface Point to Plane* and *Planar Edge to back-projected Plane* geometric constraints to estimate the extrinsic parameters of the 3D LiDAR and a stereo camera using a marker-less planar calibration target. As highlighted in the previous paragraph, each sensing modality has a different physical measurement principle; thus, sensor setups with more modalities may duplicate the calibration efforts, especially in mobile robots in which sensors are frequently dismounted or repositioned. For this reason, reference [145,148] presented a novel calibration method to extrinsically calibrate all three sensing modalities, namely radar, LiDAR, and camera with a specially designed calibration target. Table 5 below summarizes the open-source extrinsic sensor calibration tools, specifically for camera, LiDAR sensor, and radar sensor extrinsic calibration.

**Figure 9:**
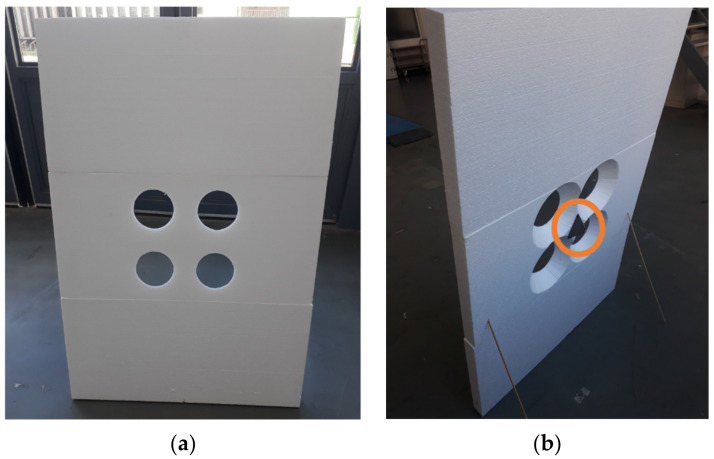
The proposed calibration target design to jointly extrinsic calibrate multiple sensors (radar, camera, LiDAR). It consists of four circulars, tapered holes centrally located within a large rectangular board at the (**a**) front of the board, and a metallic trihedral corner reflector (circled in orange) located between the four circles at the (**b**) rear of the board. Figure source from [146,147] and modified.

Reference [145] proposed a novel extrinsic calibration tool that utilizes a target-based calibration approach and a *joint* extrinsic calibration method to facilitate the extrinsic calibration of three sensing modalities. The proposed calibration target design consists of four circular, tapered holes centrally located within a large rectangular board and a metallic trihedral corner reflector located between the four circles at the rear of the board (Figure 9). The corner reflector provides a strong radar reflection as the Styrofoam board is largely transparent to electro-magnetic radiation. Additionally, the circular edges provide an accurate and robust detection for both LiDAR (especially when intersecting with fewer LiDAR beams) and camera. The authors of this system established three possible optimization configurations for joint extrinsic calibration, namely:

- Pose and Structure Estimation (PSE). It estimates the latent variables of the true board locations and optimizes the transformations to a precise estimate of all calibration target poses employing the estimated latent variables.
- Minimally Connected Pose Estimation (MCPE). It relies on a reference sensor and estimates the multi-sensing modalities transformations to a single reference frame.
- Fully Connected Pose Estimation (FCPE). It estimates the transformations between all sensing modalities “jointly” and enforces a loop closure constraint to ensure consistency.

The proposed calibration tool [146] has bindings with the commonly employed ROS middleware and provides the joint optimization configurations to estimate the sensor poses from simultaneous calibration board detection in multiple locations. It outputs a transformation matrix (*P*) that can be used to transform the detections from the *source* reference frame to *target* reference frame and the poses of the sensor with respect to the parent link for visualization (in ROS). They compared the PSE, MCPE, and FCPE joint optimization results based on multiple variables, such as the required number of calibration board locations and the MCPE reference sensor selections. The results demonstrate that the FCPE joint optimization provided better performance than both MCPE and PSE when employing more than five board locations. A detailed discussion of each joint optimization configuration and its algorithm, and the geometry of the calibration board are beyond the scope of this paper (see [146,147] for a more comprehensive overview).

**Figure 10:**
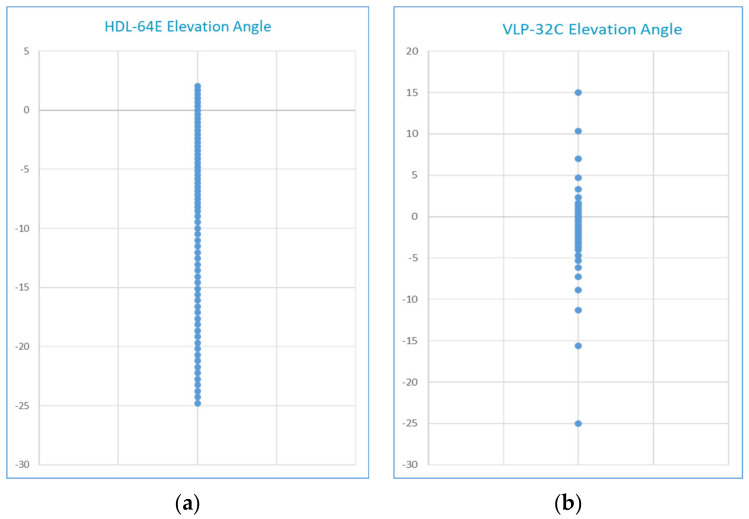
A graphical representation of the vertical laser points of the (**a**) Velodyne HDL-64E and the (**b**) Velodyne VLP-32C. Reference [145] utilizes the Velodyne HDL-64E which consists of 64 channels (layers), and the vertical laser beams are distributed uniformly across the vertical FoV between −24.9° to 2°. The initial sensor configurations employed by the current authors [22] employs the Velodyne VLP-32C which consists of 32 channels (or layers) where the vertical laser beams are concentrated in the middle of the optical center across the vertical FoV between −25° to 15°. Based on sensor user manual [68].

The current authors utilized and reviewed the calibration tool from reference [146] to extrinsic calibrate the Velodyne VLP-32C LiDAR sensor, SmartMicro UMRR-96 T-153 radar sensor, and Falcon-IQ EZIP-T030(E) Internet Protocol (IP) industrial zoom monocular camera in an initial multi-sensor setup [22]. Observations and recommendations arising from this work include:

- Ensure that the edges of the circles have sufficient contrast with the background, specifically when calibrating the cameras outdoors as was necessary in our case. Though, it is recommended in [146] that calibration of sensors be done indoors to avoid strong wind which may overturn the calibration board.
- Ensure that the camera lenses are protected from rain droplets to reduce noise when calibrating the sensors outdoors, particularly during rainy and blustery weather conditions.
- Additional or modified scripts may be required to match the ROS sensor message types of the board detector nodes depending on the employed ROS sensor drivers. For instance, a Continental ARS430 radar was utilized in [146] and exploited the AutonomouStuff-provided ROS messages which output the detections in an AutonomouStuff sensor message array format [101]. However, the ROS driver from SmartMicro radars outputs the detections in a ROS sensor message type of PointCloud2 format [113]. Table 6 summarizes the sensor message types for each board detector node (as input requirements) of the extrinsic calibration tool.
- Ensure that the edges of the four circles are detected (covered) with sufficient points within the LiDAR point cloud. We examined and compared the elevation angles of the Velodyne VLP-32C with the Velodyne HDL-64E ([162], utilized in [146]). The results indicated that the vertical laser points of HDL-64E are distributed uniformly between −24.9° to 2°. In comparison, the vertical laser points of Velodyne VLP-32C are concentrated in the middle of the optical center between −25° to 15°, as shown in Figure 10. Hence, the position and orientation of the lidar relative to the calibration board may have a significant effect on reported location of circles detected within the lidar data.
- It is suggested in [146] to position the calibration board in a spacious area and capture at least ten calibration board locations in the FoV of all sensors. However, it is not recommended to hold the calibration board, which can affect the detections of the corner reflector (by the radar sensor).
- The stereo camera employed in [146] was constructed from two monocular cameras; namely IDS Imaging UI-3060CP Rev. 2; and exploited the “stereo_image_proc” module in ROS [39] to create the disparity image of the perceived surroundings.

Based on this revision of extrinsic calibration tools available to the research community, it is noticed that most of them addressed only pairwise calibrations of two sensing modalities, with the notable exception of extrinsic calibration tool described in [145] which facilitates joint extrinsic calibration of more than two sensing modalities (radar, camera, and LiDAR) and has bindings with ROS middleware. Other open source extrinsic calibration tools include Kalibr that provides multiple camera calibration or camera-IMU extrinsic calibration and Calirad, that facilitates the extrinsic calibration and temporal calibration of the radar, camera, and LiDAR sensors. It is emphasized again that individual sensors are intrinsic calibrated before implementing extrinsic calibration.

In contrast to *target-based* extrinsic calibration methods, *targetless* extrinsic calibration approach methods estimate motion of the sensors or features in the perceiving surroundings, such as road markings to determine extrinsic calibration of the sensors.

#### 3.1.3. Temporal Calibration Overview

Temporal calibration is the process of determining the synchronicity (or relative time delay) of multiple sensor data streams with potentially different frequencies and latencies in a multi-sensor setup [119]. For instance, the camera usually captures images at 30 FPS or less, while a LiDAR sensor may scan at a rate as low as 5 Hz. One approach of synchronizing the sensor data is to establish the closest match between the message header timestamps obtained at endpoints (computer). However, in principle, synchronization based on message timestamps is suboptimal because sensors may have unknown latencies, such as communication transfer delays or pre-processing delays in the sensor circuitry [118]. These unknown latencies may not be determinable directly and will likely differ from one sensor to another. The approximate time synchronizer method in the ROS message filter module [164] matches the messages from each sensing modality (or topic in ROS term) based on their header timestamps as a means of time synchronization using an adaptive algorithm. The adaptive algorithm first determines the latest message among the heads of the topic-specific queues as a reference point, and approximately synchronize these messages based on the estimated reference point and within a given threshold.

We utilized the approximate time synchronizer method in [164] to synchronize the sensor data in an initial multi-sensor setup [22]. The results demonstrated that an average of 86.6 per cent of sensor messages with varying frequency of operation were synchronized within a threshold of 50 milliseconds. Further, the most prolonged unsynchronized periods between the *camera and LiDAR* were found to be 850 milliseconds; between *LiDAR and radar*, it was 870 milliseconds; and between *camera and radar*, it was 880 milliseconds. Another synchronization method based on messages header timestamps in ROS is the exact time synchronizer [164], which requires the incoming messages to have an exact timestamp for synchronization. A comprehensive overview of the adaptive algorithm employed in the approximate time synchronizer method and the usage of the methods are beyond the scope of this paper (see [164] for a more detailed overview).

Temporal calibration is often overlooked and is crucial in multi-sensor fusion applications, such as self-driving vehicles which must perform complex sensing and estimation tasks in real-time, such as state estimations and obstacle detections [118]. There are two approaches to temporally calibrate the sensors: *external synchronization* that utilizes external hardware for time synchronization and *internal synchronization*, exploiting the attaching timestamps on each sensor measurement for synchronization [165,166]. The external synchronization approach uses a central hardware clock as an external source of time or a reference clock to temporal-synchronize the sensors and is precisely relatable to a real-time standard such as Universal Time Coordinated (UTC) standard time. For instance, reference [167] utilizes an external Novatel SMART6-L Global Positioning System (GPS) as a reference clock and exploits the GPS timestamps information to synchronize the system (or computer) clock. Conversely, the *internal synchronization* approach synchronizes the sensors based on the associated timestamps without the external source of time to obtain a consistent view of time across all sensor networks. Reference [168] proposes the passive synchronization algorithms to determine the time offsets when the device and sensor clocks drift, and can significantly reduce the synchronization error, even in the presence of an unknown latency and for sensors with significant clock errors.

**Figure 11:**
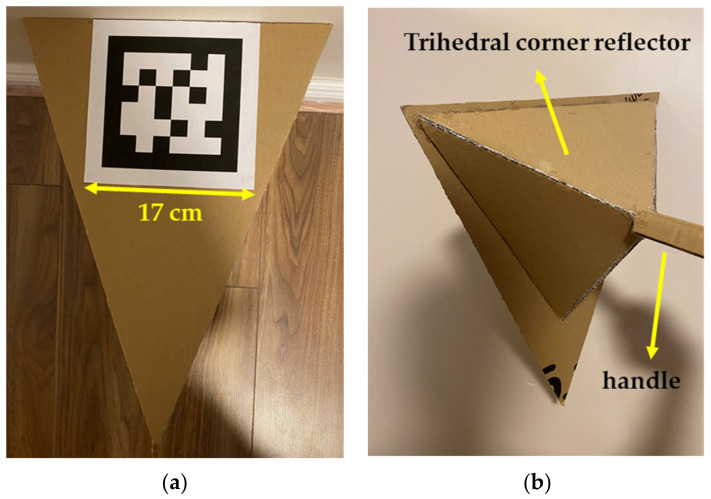
The proposed triangular calibration target design to spatial temporal calibrates the sensors (camera, radar, LiDAR). (**a**) Front view of the calibration board consists of a printed AprilTag marker with a size of approximately 17 cm in length. (**b**) The trihedral corner reflector is attached at the rear of the triangular board in which the inner sides are overlaid with aluminum foil. The calibration target in figure is constructed based on and reference [169,170,171] and through personal communication [172].

A complete sensor-to-sensor calibration, also known as the spatial-temporal calibration, involves extrinsic calibration of the sensors to a unified coordinate space and temporal calibration to estimate the relative time delays between sensor data streams. Reference [169] presents a spatial-temporal calibration method that uses the estimated continuous-time moving object trajectories from Gaussian Processes (GPs) and a target-based approach to calibrate the sensors relative to one another. It utilizes estimated object velocities to estimate relative time delays between sensors. These [169] experiments demonstrated that the proposed algorithm could reliably determine the time delays up to a fraction of the fastest sensor sampling rate. The implementation of the method proposed in [169] has been open sourced in [170] and it has bindings with ROS middleware. Additionally, it applies to any multi-sensor setup once the employed multi-sensor can determine the 3D position of a moving “target”. An insightful discussion of the employed GP algorithms is beyond the scope (see [169,170,171] for a more comprehensive overview). Further, through personal communication, the target detections become unstable from six meters or more depending on the tracker size. The materials from which the calibration tracker is constructed include (Figure 11) [172]:

- Styrofoam or cardboard to fabricate the triangular planar pattern,
- Printed AprilTag marker with a size of approximately 17 cm in length, located at the front of the triangular planar and,
- Cardboards to assemble a trihedral corner reflector where the three inner sides of the reflector are overlaid with aluminum foil and attached at the rear of the triangular planar.

Other spatial-temporal calibration methods include employing a target-based approach and the spatial-temporal relationships of the target measurements (positions) to estimate the time delays and the sensors extrinsic parameters [173]. In [174], the PolySync bus (external hardware) was employed to publish a synchronized timestamp based on the IEEE 1588 Precision Time Protocol (PTP), to all computers as a means of time synchronization during the data acquisition process.

To summarize, estimating the time delays between multiple sensors operating at different frequencies is vital, especially in time-critical autonomous systems, to precisely perform autonomous tasks in real-time, such as obstacles detection, and vehicle state estimation, and ultimately to prevent collisions.

### 3.2. Sensor Fusion

Sensor fusion is an essential aspect of most autonomous systems, e.g., on-road self-driving cars and autonomous Unmanned Ground Vehicles (UGV). It integrates the acquired data from multiple sensing modalities to reduce the number of detection uncertainties and overcome the shortcomings of individual sensors operating independently. Moreover, sensor fusion helps to develop a consistent model that can perceive the surroundings accurately in various environmental conditions [175]. For instance, camera and radar fusion may provide high-resolution images and the relative velocities of the detected obstacles in the perceived scene. Table 7 below qualitatively summarizes the strengths and weaknesses of the commonly utilized perception-based sensors in AVs based on their technical characteristics and other external factors, such as weather and illumination conditions.

The research on multi-sensor fusion systems in AVs for environment perception and object detection is well-established in the literature [19,21,30,167,176,177,178]. Presently, three primary sensor combinations for obstacle detection are prevalent in the literature, including *camera-LiDAR* (CL); *camera-radar* (CR); and *camera-LiDAR-radar* (CLR) sensor combinations. A survey conveyed by [21] showed that the CR sensor combination is the most employed in the multi-sensor fusion systems for environment perception, followed by CLR and CL. The CR sensor combination offers high-resolution images while obtaining additional distance and velocity information of surrounding obstacles. For instance, Tesla utilized the CR sensor combination and other sensors, such as ultrasonic sensors, to perceive vehicle surroundings [8]. Similarly, the CLR sensor combination can provide resolution at a greater range, and precisely understands the surroundings through the LiDAR point clouds, and depth map information. It also improves the safety redundancy of the overall autonomous system. For instance, Waymo and Navya [179] used the CLR sensor combination for environment perception in their AVs.

#### 3.2.1. Sensor Fusion Approaches

There are three primary approaches to combine sensory data from various sensing modalities in the MSDF frameworks: *high-level fusion* (HLF), *low-level fusion* (LLF), and *mid-level fusion* (MLF) [180]. In the HLF approach, each sensor carries out object detection or a tracking algorithm independently and subsequently performs fusion. For instance, reference [30] utilized the HLF approach to fuse the processed data, i.e., radar signals and LiDAR point clouds independently and subsequently used a *non-linear Kalman Filter* method to detect obstacles and state tracking. The HLF approaches are often adopted due to a lower relative complexity than the LLF and MLF approach. However, HLF provides inadequate information as classifications with a lower confidence value are discarded if, for example, there are several overlapping obstacles.

Contrarily, with the LLF approach, data from each sensor are integrated (or fused) at the lowest level of abstraction (raw data). Therefore, all information is retained and can potentially improve the obstacle detection accuracy. Reference [181] proposed a two-stage 3D obstacle detection architecture, named *3D-cross view fusion* (3D-CVF). In the second stage, they utilized the LLF approach to fuse the joint camera-LiDAR feature map obtained from the first stage with the low-level camera and LiDAR features using a *3D region of interest (RoI)-based pooling* method. They evaluated the proposed method on KITTI and nuScenes datasets and reported that the object detection results outperformed the state-of-the-art 3D object detectors in the KITTI leaderboard (see reference [181] for a more comprehensive summary). In practice, the LLF approach comes with a multitude of challenges, not least in its implementation. It requires precise extrinsic calibration of sensors to accurately fuse their perceptions of the environment. The sensors must also counterbalance ego-motion (3D motion of a system within an environment) and be temporally calibrated [180].

The MLF, otherwise known as feature-level fusion, is an abstraction level between LLF and HLF. It fuses multi-target features extracted from the corresponding sensor data (raw measurements), such as color information from images or location features of radar and LiDAR, and subsequently perform recognition and classification on the fused multi-sensor features. Reference [182] proposed a feature-level sensor fusion framework to detect targets in a dynamic background environment with limited communication capability. They utilized the *Symbolic Dynamic Filtering* (SDF) algorithm to extract the low-dimensional features from multiple infrared sensors in different orientations and in the presence of changing ambient light intensities and subsequently fusing the extracted features as clusters with the *agglomerative hierarchical clustering* algorithm for moving target detection. The MLF, however, appears to be insufficient to achieve a SAE Level 4 or Level 5 AD system due to its limited sense of the environment and loss of contextual information [183].

#### 3.2.2. Sensor Fusion Techniques and Algorithms

Sensor fusion techniques and algorithms have been extensively studied over the last number of years and now, are well-established in the literature. However, a recent study [184,185] revealed that obtaining the current state-of-the-art fusion techniques and algorithms is an arduous and challenging task due to multidisciplinary and variants of proposed fusion algorithms in the literature. The study of [19] classified these techniques and algorithms into classical sensor fusion algorithms and deep learning sensor fusion algorithms. On the one hand, the classical sensor fusion algorithms, such as knowledge-based methods, statistical methods, probabilistic methods, et cetera, utilize the theories of uncertainty from data imperfections, including inaccuracy and uncertainty to fuse sensor data. Reference [186] proposes a real-time roundabout detection and navigation system in a road environment utilizing a combination of the proposed “Laser Simulator” algorithm to detect objects and the knowledge-based fuzzy logic (FL) algorithm for decision making.

On the other hand, the deep learning sensor fusion algorithms involve generating various multi-layer networks that enable them to process raw data and extract features to perform challenging and intelligent tasks, e.g., object detection in an urban environment for AV. In the AV context, algorithms, such as Convolutional Neural Network (CNN) and Recurrent Neural Network (RNN) are among the most employed algorithms in perception systems. Reference [187] proposed an advanced weighted-mean You Only Look Once (YOLO) CNN algorithms to fuse RGB camera and LiDAR point cloud data to improve the real-time performance of object detection. YOLO detector was first created in 2016 by [188] and has achieved a significant milestone over the last number of years. It is a single-stage detector that predicts bounding boxes and produces class probabilities with confidence scores on an image in a single neural network (one evaluation only). The YOLO based model provides fast detection speed of 45 FPS with 59.2% average precision (AP, an evaluation metric that measures object detection or information retrieval model performances) on the VOC 2007 dataset [188]. Besides, the latest YOLOv4 released by [189] in April 2020, achieves state-of-the-art results at a real-time speed on the MS COCO dataset of approximately 65 FPS with 43.5% AP (and 65.7% AP50—IoU above 50%) on an NVIDIA^®^ Tesla^®^ V100 Graphical Processing Unit (GPU). In [190], the authors proposed a CNN-based method to detect aggressive driving behaviors through emotions using near-infrared light and thermal cameras. They conducted score-level fusion using the CNN output scores from near-infrared light images and thermal images to improve the detection accuracy. Their proposed method achieved a high classification accuracy of emotions and demonstrated that their proposed technique achieved better performance than the conventional methods for emotion detection.

In addition, with the advent of 3D sensors and diverse applications for understanding the 3D environment of the surrounding AV, there is an increased research focus on 3D object detection. Reference [191] leverages their previously proposed *VoxelNet* framework in [192] and presented two feature-level fusion approaches called PointFusion and VoxelFusion to combine the RGB and point cloud data for 3D object detection. According to [192], VoxelNet is a generic 3D object detection network that unifies feature extraction and bounding box prediction processes into a single stage, end-to-end trainable deep network. The PointFusion method uses the known calibration matrix to project 3D points onto the image and, subsequently extracts image features from a pre-trained 2D CNN and concatenate them at the point level. Subsequently, they leveraged the VoxelNet architecture to process the concatenated features and the corresponding points jointly. In contrast, the VoxelFusion method projects the non-empty 3D voxels created by the VoxelNet onto the image and extract features within the 2D ROIs and consequently concatenates the poo-led image features at the voxel level.

Reference [193] presented a *PointFusion* framework that leverages the image data and raw point cloud data for 3D object detection. They utilized the CNN and PointNet [194] architectures to process the image and point cloud independently and subsequently combine the resulting outputs to predict multiple 3D box hypothesis and their corresponding confidences. The PointNet architecture is a novel neural network that provides a unified architecture for applications ranging from 3D classification to scene semantic parsing for processing raw point cloud data. Other deep learning-based sensor fusion algorithms, to name a few, include:

- **ResNet**, or Residual Networks, is a residual learning framework that facilitates deep networks training [195].
- **SSD**, or Single-Shot Multibox Detector, is a method that discretizes bounding boxes into a set of boxes with different sizes and aspect ratios per feature map location to detect objects with variant sizes [196]—it overcomes the limitation of YOLO small and variant-scale object detection accuracy.
- **CenterNet** [197] represents the state-of-the-art monocular camera 3D object detection algorithm, which leverages key-point estimation to find center points of bounding boxes and regresses the center points to all other object properties, including size, 3D location, orientation, and pose.

Table 8a below summarizes the strengths and weaknesses of the sensor fusion approaches: HLF, LLF, and MLF, and presents an overview of the sensor fusion techniques and algorithms for obstacle detection, namely YOLO, SSD, VoxelNet, and PointNet, in Table 8b. The readers interested in detailed discussions about sensor fusion techniques and algorithms for various applications ranging from perception, including 2D or 3D obstacle detection and lane tracking, to localization and mapping are advised to refer to [19,20,23,24,25,184,191,192,193,194,195,196,197,198,199,200,201,202,203,204,205,206].

#### 3.2.3. Challenges of Sensor Fusion for Safe and Reliable Environment Perception

Undoubtedly, the multi-sensor fusion technologies, based on extensive research, have achieved relatively comprehensive advantages in autonomous systems ranging from humanoid robots to AVs. These systems are often equipped with an array of sensors that could generate a large volume of data per hour. For instance, an AV could generate approximately 383 GB to 5.17 TB (Terabyte) of data per hour [207]. Therefore, it requires large computational power to process these data. Reference [208] reviewed the computing platform implementation of an SAE Level 4 AV from a leading autonomous driving company and examined several existing processing solutions for AD. In addition, they presented and prototyped an AD computing architecture and software stack that is secure, modular, dynamic, energy-efficient, and high performance. Their prototype system consumes an average of 11 Watt (W) of power and can drive a mobile vehicle at 8 km per hour, using an ARM Mobile System on Chip (SoC). From the software perspective, combining reinforcement learning (RL) techniques with supervised learning algorithms could help to reduce computational power, training data requirements, and training time.

RL is a machine learning (ML) method that uses the feedback from their actions and experiences to train ML models in an interactive environment. In contrast, supervised learning algorithm utilizes labelled data to train ML models (refer to reference [25] for a more detailed overview). However, it is challenging to train and annotate data from all possible scenarios, including but not limited to location, terrain, and weather, which an AV may encounter in the real-world. Although collaboration and sharing of data could benefit the development of autonomous systems, it is unlikely as companies researching autonomous systems are unwilling to share resources due to the fear of diluting their competitive advantage [25,209]. Additionally, the performance of an ML/DL for object detection and localization and mapping are influenced by the employed dataset’s quality; hence, poor data quality could lead to the proverbial “garbage-(data)-in and garbage-(data)-out”. The founder and CTO of Roboflow wrote that 33% out of 15,000 samples (or images) in Udacity Dataset 2 are not annotated and the annotated bounding boxes (or objects of interest) are oversize [210].

The functional safety of the utilized DL models in multi-sensor AVs can also be a challenge due to the opaque nature of DL algorithms. Reference [25] highlighted that it is critical to further research the available safety validation methods and the interpretability of neural networks before deploying DL models on the road. In addition, autonomous systems that utilize DL architectures are vulnerable to adversarial attacks. The attackers overlaid typical images with adversarial samples (or perturbed images) that represent subtle changes to the inputs of the DL systems but resulted in misclassification of objects with high confidence scores [25]. Other sensor fusion challenges include biases in collected datasets, overfitting of training datasets, imprecision, and uncertainty in the data measurements, such as noise relating to calibration errors, quantization errors, loss of precisions, missing values, et cetera. Transforming multi-sensor data into a standard frame of reference may also pose a challenge in sensor fusion implementations.

From an environmental perspective, one of the remaining challenges of sensor fusion for reliable and safe perception is the performance of vision sensors in harsh weather conditions such as snow, fog, sandstorms, or rainstorm. Such conditions can impact the vision and range measurements of vision sensors, leading to a decrease in visibility distance and resulting in erroneous and misleading outputs. In a worst-case scenario, sensors may experience a partial or complete sensor failure, which can be disastrous for AVs and their surroundings. Hence, based on learned experiences and historical data, it is important to evaluate the risk of failure early in the process and enable drivers to interrupt or completely disengage the autonomous systems [19].

In general, quality data is the key to a safe and reliable environment perception. DL/ML models employ these data to learn about the environment’s features and perform object detection. Thus, it is essential to cleanse and pre-process the data before implementing DL/ML algorithms. However, DL algorithms are prone to malicious attacks, which can be disastrous in safety-critical systems, such as AVs. Further research and extensive testing of autonomous systems are essential to assess all possible solutions to prevent malicious attacks and evaluate all possible sensors and system failure risks and alternative solutions in the case of sensors or system failures. A detailed discussion about the sensor fusion challenges, including adversarial attacks and possible preventions is beyond the scope of this paper (see [16,19,25,211,212,213,214] for a more comprehensive overview).

## 4. Conclusions and Future Research Recommendations

In this paper, we presented a complete overview of the perception block in the AD systems. We surveyed the technical performance and capabilities of sensors from various manufacturers in different conditions, mainly focusing on vision cameras, LiDAR sensors, and radar sensors. We also presented an overview of the three main categories of sensor calibration, which may be considered a foundation block of any autonomous systems and summarize the existing open-source multi-sensor calibration packages that can calibrate multiple sensors simultaneously. Finally, we reviewed some of the fusion algorithms that were successfully established in the literature and highlighted some of the challenges in the sensor fusion field and possible future research directions for AD systems.

The area of AVs is vast and consists of a wide range of technical disciplines and technologies, from electronics, sensors, and hardware to algorithms for vehicle state control and decision-making, and economic, legal, and social aspects. Sensors are elementary to the perception of surroundings, localization and mapping, and vehicle state control. Currently, AVs primarily incorporate multiple, complementary sensors, such as IMUs, radars, LiDARs, and cameras to overcome the limitations of individual sensors operating independently.

It is essential to calibrate sensors before the implementation of algorithms for processing data. A precise sensor calibration allows the AV to understand its position and orientation in the real-world coordinates. We examined the three main categories of sensor calibration, each of which is necessary: namely, intrinsic calibration, extrinsic calibration, and temporal calibration and related algorithms. Additionally, we provided a comparative overview of several existing open-source calibration packages that have been successfully employed in recent research. It is apparent that most existing open-source calibration tools for extrinsic and temporal calibration only address pairwise calibration of a maximum of two sensing modalities.

The approaches to sensor calibration in recent studies focus on offline methods to calibrate the sensors. The offline method to sensor calibration utilizes the specially designed calibration targets to provide accurate calibration results, but it is not flexible. For instance, the vehicle is required to recalibrate if there is a geometry change between the sensors. Moreover, external factors, such as temperature and vibrations, may affect the calibration accuracy as multi-sensor are commonly factory calibrated. Therefore, it is critical to further research online and offline calibration techniques to automatically detect and refine calibration parameters to provide precise estimation of the presence and position of objects in autonomous operation.

The development of reliable and efficient obstacle detection in self-driving vehicles is critical to achieving autonomous driving. The practical approach in recent studies for safe and reliable obstacle detection is to combine information from multimodal sensors, such as distance information, velocity, color distribution, et cetera, to provide accurate, robust, and reliable detection results. We reviewed the three primary approaches of sensor fusion: namely high-level fusion, mid-level fusion, and low-level fusion and subsequently reviewed recently proposed multi-sensor fusion techniques and algorithms for obstacle detection. Similarly, we highlighted several challenges of multi-sensor fusion for reliable and safe environment perception. The main challenges are environmental conditions, invulnerability to malicious attacks in DL models, poor quality datasets, or datasets not addressing all possible environments for the AV, and the computation cost to process large volumes of datasets in real-time. Therefore, companies and researchers must evaluate the risk of failure and implement alternative solutions for drivers to handle worst-case scenarios.

Further developments to improve object detection performance in all possible scenarios, including harsh weather conditions, are essential to providing safe and reliable scene perception. It is critical to developing accurate, robust, and reliable object detection algorithms that can distinguish obstacles against the environment. One approach to providing a more reliable and accurate obstacle detection is to enhance existing sensor fusion algorithms through deep learning approaches or deep reinforcement learning approaches [215]. Another approach would be to invest in sensors hardware technology to provide a higher resolution of the surroundings [19].

## Figures and Tables

**Table 1:** Summary of recent studies on sensor and sensor fusion technologies.

Reference	Summary
Velasco-Hernandez et al. [15]	An overview of the AD architectures—technical and functional architectures depending on the domain of their definition. Further, the authors highlight the perception stage of self-driving solutions as a component, detailing the sensing component and sensor fusion techniques to perform localization, mapping, and obstacle detection.
Fayyad et al. [19]	An overview of the state-of-the-art deep learning sensor fusion techniques and algorithms for perception, localization, and mapping.
Campbell et al. [20]	A summary of sensor technologies, including their strengths and weaknesses, that were commonly used to develop an autonomous vehicle. Moreover, the authors examined some of the sensor fusion techniques that can be employed in both indoor and outdoor environments, and algorithms for obstacle detection, navigation, and environment modelling.
Wang et al. [21]	A discussion of sensor technology and their performance in various conditions. The authors surveyed and presented a detailed summary of the multi-sensor fusion strategies in recent studies and techniques to establish motion model and data association in multi-target tracking.
Yeong et al. [22]	A summary of advantages and disadvantages of perception-based sensors and the architecture of multi-sensor setup for obstacle detection in industrial environments. Moreover, the authors highlighted some of the challenges to temporal synchronize multiple data streams in AD applications.
Jusoh, S. & Almajali, S. [23]	A discussion of the current state-of-the-art multi-sensor fusion techniques and approaches for various applications such as obstacle detection, localization, and mapping, in three major domains, namely robotics, military, and healthcare.
Castanedo, F. [24]	A discussion of the classification of data fusion techniques based on several criteria and providing a comprehensive overview of the most employed methods and algorithms for data association, state estimation, and decision fusion tasks.
Kuutti et al. [25]	An overview of deep learning approaches and methods for autonomous vehicle control, and the challenges to deep learning-based vehicle control. The authors considered these approaches for three categories of tasks: lateral (steering), longitudinal (acceleration and braking), and simultaneous lateral and longitudinal control, and discussed the relevant methods in detail.
Hu et al. [26]	A discussion of the perception-based sensors for intelligent ground vehicles in off-road environment and a comprehensive review of the current state-of-the-art multi-sensor fusion approaches. In addition, the author summarized the main considerations of on-board multi-sensor configurations and reviewed the architectural structure of perception systems and applications for obstacle detection in diverse environments.

**Table 2:** General specifications of stereo cameras from various manufacturers that we reviewed from our initial findings. The acronyms from left to right (in second row) are horizontal field-of-view (HFOV); vertical field-of-view (VFOV); frames per second (FPS); image resolutions in megapixels (Img Res); depth resolutions (Res); depth frames per second (FPS); and reference (Ref). The “-” symbol in table below indicates that the specifications were not mentioned in product datasheet.

								Depth Information			
	Model	Baseline (mm)	HFOV (°)	VFOV (°)	FPS (Hz)	Range (m)	Img Res (MP)	Range (m)	Res (MP)	FPS (Hz)	Ref
Roboception	RC Visard 160	160	61 *	48 *	25	0.5–3	1.2	0.5–3	0.03–1.2	0.8–25	[40,41]
Carnegie Robotics^®^	MultiSense™ S7 ^1^	70	80	49/80	30 max	-	2/4	0.4 min	0.5–2	7.5–30	[40,42,43]
	MultiSense™ S21B ^1^	210	68–115	40–68	30 max	-	2/4	0.4 min	0.5–2	7.5–30	[40,44]
Ensenso	N35-606-16-BL	100	58	52	10	4 max	1.3	-			[40,45]
Framos	D435e	55	86	57	30	0.2–10	2	0.2 min	0.9	30	[40,46]
Nerian	Karmin3 ^2^	50/100/250	82	67	7	-	3	0.23/0.45/1.14 min	2.7	-	[40,47]
Intel RealSense	D455	95	86	57	30	20 max	3	0.4 min	≤1	≤90	[40,48]
	D435	50	86	57	30	10 max	3	0.105 min	≤1	≤90	
	D415	55	65	40	30	10 max	3	0.16 min	≤1	≤90	
Flir^®^	Bumblebee2 ^3^	120	66	-	48/20	-	0.3/0.8	-			[40,49]
	Bumblebee XB3 ^3^	240	66	-	16	-	1.2				[50,51]

^1^ HFOV, VFOV, image resolutions, image frame rates and depth information depend on the variant of focal length (optical lens geometry). ^2^ Specifications stated are in full resolution and monochrome, focusing on the standard 4 mm lens. ^3^ Offers either 2.5 mm, 3.8 mm or 6 mm lenses (specifications focus on 3.8 mm lens) but product no longer being produced or offered (discontinued). * A 6 mm lens has a HFOV of 43° and a VFOV of 33°.

**Table 4:** Summary of the general specifications of radar sensors from SmartMicro, Continental and Aptiv Delphi. The acronyms (first column from top to bottom) are frequency (Freq), horizontal FoV (HFOV), vertical FoV (VFOV), range accuracy (Range Acc), velocity range (Vel Range), input/output interfaces (IO Interfaces) and ROS (Robotic Operating System) drivers for that specific sensors. The “-” symbol in table indicates that the specifications were not mentioned in product datasheet.

	Aptiv Delphi		Continental	SmartMicro
	ESR 2.5	SRR2	ARS 408-21	UMRR-96 T-153 ^1^
**Freq (GHz)**	76.5	76.5	76…77	79 (77…81)
HFOV (°)		±75		
Short-Range			±9	≥130
Mid-Range	±45			≥130
Long-Range	±10		±60	≥100 (squint beam)
VFOV (°)	4.4	10	20	15
Short-Range			14	
Long-Range				
Range (m)	1–60	0.5–80 ^2^		
Short-Range	1–175 ^2^		0.2–70/100	0.15–19.3 ^3^
Mid-Range				0.4–55 ^3^
Long-Range			0.2–250	0.8–120 ^3^
Range Acc (m)	-	±0.5 noise and ±0.5% bias	-	
Short-Range				<0.15 or 1% (bigger of)
Mid-Range				<0.30 or 1% (bigger of)
Long-Range				<0.50 or 1% (bigger of)
Vel Range (km/h)	-	-	-400…+200 ^4^	
Short-Range				−400…+140 ^4^
Mid-Range				−340…+140 ^4^
Long-Range				−340…+140 ^4^
IO Interfaces	CAN/Ethernet ^5^	PCAN	CAN	CAN/Automotive Ethernet
ROS Drivers	[101,102]		[103]	[104]
**Reference**	[51,105,106,107,108,109]		[110,111,112]	[113]

^1^ It is recommended to use PCAN-USB adapter from PEAK System for connections of Controller Area Network (CAN) to a computer via Universal Serial Bus (USB) [114]. ^2^ Range indicated for ESR 2.5 (long-range mode) and SRR2 is measured at 10dB and 5 dB, respectively. ^3^ Range may vary depending on the number of targets in the observed environment and will not achieve a 100% true-positive detection rate. ^4^ A negative velocity range indicates the object is moving away from the radar (opening range) and a positive value indicates the object is moving toward the radar (closing range) [115]. ^5^ Internet Protocol (IP) address specified on request with a sale unit and is not modifiable by user [116].

**Table 5:** An overview of the available open-source extrinsic sensor calibration tools for multi-sensing modalities, specifically for LiDAR, radar, stereo camera, and monocular camera. The acronyms of the columns (from left to right) are the referenced literature (Ref), stereo camera (S), monocular camera (M), LiDAR (L) and Radar (R). The platform and toolbox column refer to the working environment of the toolbox and a reference link to the open-source calibration toolbox. Further, the calibration target column summarizes the calibration target used for extrinsic sensor calibration. The symbols ✓ and ✖ indicate whether the proposed open-source toolbox can calibrate a particular sensor. The “*” symbol indicates that the proposed calibration tool claims to support monocular camera calibration. The “~” symbol indicates that a stereo camera could be calibrated as two separate monocular cameras, but in principle, it is suboptimal. The “-“ symbol indicates that the extrinsic calibration tool is not mentioned or openly or freely available to the research community. Based on [145] with modification.

Ref	S	M	L	R	Platform	Toolbox	Calibration Target
[145] ^1^	✓	*	✓	✓	ROS	[146]	Styrofoam planar with four circular holes and a copper plate trihedral corner reflector.
[148]	~	✓	✓	✓	-	-	Checkerboard triangular pattern with trihedral corner retroreflector.
[152]	✓	✖	✓	✖	MATLAB	[153]	LiDARTag ^2^ and AprilTag ^2^.
[154] ^3^	✓	*	✓	✖	ROS	[155]	Planar with four circular holes and four ArUco markers ^4^ around the planar corners.
[156]	✓	*	✓	✖	ROS	[157]	ArUco marker on one corner of the hollow rectangular planar cardboard marker.
[143]	~	✓	✓	✖	ROS	[158]	3D marker with four circular holes pattern.
[159]	~	✓	✓	✖	ROS	[160]	Planar checkerboard pattern.

^1^ The toolbox binds with the commonly employed ROS and includes a monocular camera detector for extrinsic calibration, but reported results relate to stereo camera only [145]. ^2^ LiDARTag (point clouds) and AprilTag (images) is a visual fiducial tag (QR-code like pattern). ^3^ The extrinsic calibration tool is an enhancement version of the previous work from [161]. ^4^ ArUco marker is a synthetic 2D square marker with a wide black border and an inner binary matrix.

**Table 6:** An overview of the ROS topic message types as input requirements for each calibration board detector node, namely monocular camera detector (mono_detector), LiDAR detector (lidar_detector), stereo camera detector (stereo_detector), and radar detector (radar_detector). Based on reference [145,152]. A detailed overview of the ROS sensor message types is available in reference [163].

Detector	Subscribed Topic Name	ROS Sensor Message Types
LiDAR	/velodyne_points	sensors_msgs::PointCloud2
Stereo	/ueye/left/image_rect_color /ueye/left/camera_info /ueye/right/camera_info /ueye/disparity	sensor_msgs::Image sensor_msgs::CameraInfo sensor_msgs::CameraInfo stereo_msgs::DisparityImage
Monocular	/ueye/left/image_rect_color /ueye/left/camera_info	sensor_msgs::Image sensor_msgs::CameraInfo
Radar	/radar_converter/detections	radar_msgs::RadarDetectionArray ^1^

^1^ AutonomouStuff, an automotive platform that offers solutions for developing and deploying AD applications, who provide the generic radar output messages that are not currently available in the commonly employed ROS sensor messages module (deprecated in latest version of ROS1).

**Table 7:** A comparison of the commonly employed sensors in self-driving cars; camera, LiDAR, and radar, based on technical characteristics and other external factors. The “✓” symbol indicates that the sensor operates competently under the specific factor. The “~” symbol indicates that the sensor performs reasonably well under the specific factor. The “✖” symbol indicates that the sensor does not operate well under the specific factor relative to the other sensors.

Factors	Camera	LiDAR	Radar	Fusion
Range	~	~	✓	✓
Resolution	✓	~	✖	✓
Distance Accuracy	~	✓	✓	✓
Velocity	~	✖	✓	✓
Color Perception, e.g., traffic lights	✓	✖	✖	✓
Object Detection	~	✓	✓	✓
Object Classification	✓	~	✖	✓
Lane Detection	✓	✖	✖	✓
Obstacle Edge Detection	✓	✓	✖	✓
Illumination Conditions	✖	✓	✓	✓
Weather Conditions	✖	~	✓	✓

**Table 8:** (**a**) A comparative overview of the sensor fusion approaches, namely high-level fusion (HLF), low-level fusion (LLF), and mid-level fusion (MLF) [30,180,204,205,206]. (**b**) Table below summarizes some of the sensor fusion techniques and algorithms that were successfully established in the art for obstacles detection, namely YOLO, SSD, VoxelNet, and PointNet. Further, table below presents a summary of the advantages and drawbacks of each algorithm.

(a)			
Sensor Fusion Approaches	Descriptions	Strengths	Weaknesses
High-Level Fusion (HLF)	Each sensor carries out detection or tracking algorithm separately and subsequently combines the result into one global decision.	Lower complexity and requires less computational load and communication resources. Further, HLF enables standardizing the interface towards the fusion algorithm and does not necessitate an in-depth understanding of the signal processing algorithms involved.	Provides inadequate information as classifications with a lower confidence value are discarded. Furthermore, fine-tuning the fusion algorithms has a negligible impact on the data accuracy or latency.
Low-Level Fusion (LLF)	Sensor data are integrated at the lowest level of abstraction (raw data) to be of better quality and more informative.	Sensor information is retained and provides more accurate data (a lower signal-to-noise ratio) than the individual sensors operating independently. As a result, it has the potential to improve the detection accuracy. In addition, LLF reduces latency where the domain controller does not have to wait for the sensor to process the data before acting upon it. This can help to speed up the performance—of particular importance in time-critical systems.	Generates large amount of data that could be an issue in terms of memory or communication bandwidth. Further, LLF requires precise calibration of sensors to accurately fuse their perceptions and it may pose a challenge to handle incomplete measurements. Although multi-source data can be fused to the maximum extent, there is data redundancy, which results in low fusion efficiency.
Mid-Level Fusion (MLF)	Extracts contextual descriptions or features from each sensor data (raw measurements) and subsequently fuses the features from each sensor to produce a fused signal for further processing.	Generates small information spaces and requires less computation load than LLF approaches. Further, MLF approach provides a powerful feature vector and the features selection algorithms that detect corresponding features and features subsets can improve the recognition accuracy.	Requires large training sets to find the most significant feature subset. It requires precise sensor calibration before extracting and fusing the features from each sensor.
**(b)**			
**Algorithms**	**Descriptions**	**Advantages and Drawbacks**	**Reference**
YOLO	You Only Look Once (YOLO) is a single-stage detector, which predicts bounding boxes and produces class probabilities with confidence scores on an image in a single CNN ^1^.	- Provides real-time detections. - Less accurate than SSD. - Poor detection of dense obstacles, e.g., flocks of birds, because each grid can propose only 2 bounding boxes. - Poor detection of small obstacles. - High localization error.	[19,187,188]
SSD	Single-Shot Multibox Detector (SSD) is a single-stage CNN detector that discretizes bounding boxes into a set of boxes with different sizes and aspect ratios to detection obstacles with variant sizes.	- Provides real-time and accurate obstacle detections. - Pose a challenge to detect smaller obstacles but performs better than YOLO. - Poor extractions of features in shallow layers. - Loss of features in deep layers.	[19,196,200]
VoxelNet	A generic 3D obstacle detection network that unifies feature extraction and bounding boxes prediction into a single-stage, end-to-end trainable deep network. In other words, VoxelNet is a voxelized method for obstacle detection using point cloud data.	- Does not require to extract features manually. - Requires large volume of data and memory for training.	[192,202]
PointNet	Presents a permutation-invariant deep neural network which learns global features from unordered point clouds (two-stage detection).	- Able to handle point clouds in any order, e.g., permutation independence on the order of point clouds. - Difficult to generalize to unseen point configurations.	[194,202]

^1^ CNN, or Convolutional Neural Network, is a specialized neural network that is used to process data that has an input shape like a 2D matrix, such as images.

## Data Availability

No new data were created or analyzed in this study. Data sharing is not applicable to this article.
